# A novel 5-Plex qPCR-HRM assay detecting human diarrheal parasites

**DOI:** 10.1186/s13099-020-00365-6

**Published:** 2020-05-29

**Authors:** Aline Lamien-Meda, Renate Schneider, Julia Walochnik, Herbert Auer, Ursula Wiedermann, David Leitsch

**Affiliations:** Institute for Specific Prophylaxis and Tropical Medicine, Center for Pathophysiology, Infectiology and Immunology, Medical University of Vienna, Kinderspitalgasse 15, 1090 Vienna, Austria

**Keywords:** Diarrhea, qPCR-HRM, *Dientamoeba*, *Entamoeba*, *Cryptosporidium*, *Blastocystis*, *Giardia*

## Abstract

**Background:**

Intestinal parasitic diseases occur worldwide, and their diagnosis poses considerable challenges. *Cryptosporidium* spp., *Entamoeba histolytica, Giardia intestinalis,* (and, arguably, *Dientamoeba fragilis* and *Blastocystis* spp.) are among the most important and common parasitic protozoans causing diarrhea. Several multiplex real-time PCR assays have been developed for the synchronous detection of these parasites. However, most assays include the use of hydrolysis probes, increasing the cost of stool examination. In this study, we designed and evaluated a real-time PCR protocol, based on high-resolution melting (HRM) curve analysis, to simultaneously detect and differentiate five gastrointestinal parasites.

**Results:**

Using a blinded panel of 143 clinical samples with laboratory diagnostic data to evaluate the method, we obtained a 95.8% concordance with conventional methods. Moreover, 4.2% of the samples were positive for *D. fragilis* and 2.8% additional *Cryptosporidium* infections were found with our multiplex assay. Our method is sensitive and specific for the selected parasites with the additional possibility of being run in single-plex as a backup control for mixed infections.

**Conclusions:**

The assay is a convenient and cost-effective method that could contribute to a quicker and accurate diagnosis as well as to more targeted therapies of parasite-derived diarrhea. Finally, this new multiplex PCR assay could also be instrumental in epidemiology studies on these parasites.

## Background

Human intestinal protozoan infections can lead to significant morbidity and mortality when not diagnosed timely and treated appropriately. Such infections are typically more associated with low-income countries. But with worldwide traveling and migration increasing in importance, there is also a continuous increase in the number of cases in industrialized countries. The diagnosis of intestinal protozoan diseases has remained a challenge, especially in low-endemicity countries where practitioners and health institutions are less familiar with the respective causative agents [1].

*Cryptosporidium* spp., *Entamoeba histolytica,* and *Giardia intestinalis* are the most important parasitic protozoans causing diarrhea [2, 3]. Giardiasis and cryptosporidiosis are major causes of moderate to severe diarrhea in both, developing and developed countries, leading to considerable mortality worldwide [2, 3]. Amoebiasis is the third most frequent cause of death from protozoan parasitic diseases, with high prevalence in developing countries [4, 5].

*Blastocystis* spp. and *Dientamoeba fragilis* are very common protozoan commensals whose pathogenic potential is still under evaluation [6]. Human infection rates with *Blastocystis* spp. can range between 1–60% and can even go up to 100%, depending on the geographic distribution and also the economic status of a given country [10, 11]. *D. fragilis* is a trichomonad parasite [4] of the human gastrointestinal tract and has, since 1919, been repeatedly reported in symptomatic patients [6]. The incidence of *D*. *fragilis* is equal to or even exceeds that of *G*. *intestinalis* [7].

As several intestinal protozoans can cause diarrhea, it is important to use diagnostic methods capable of easily and accurately identifying the infectious agents. A multiplex PCR approach can address challenges as presented by microscopy and immunofluorescence assays, such as the dependence on well-trained microscopists, and time or sensitivity issues. Despite the limitations, microscopy is still considered as the reference standard method for routine diagnostics due to its low cost and practicability in endemic countries. The main benefits of a multiplex PCR include cost-effectiveness, time-saving, and higher throughput. Nevertheless, the design and evaluation of a multiplex assay require an investment in terms of time and costs for optimization and validation.

In this study, we established a cost-effective and practical tandem multiplex—high resolution melting analysis for simultaneous detection and quantification of five intestinal protozoa: *Cryptosporidium* spp., *E. histolytica*, *Giardia intestinalis* (assemblages A and B), *Blastocystis* spp., and *D. fragilis*.

## Results

### Assay design and optimization

The individual primer sets used for the multiplex PCR were designed based on sequences obtained from the literature for the generation of long fragments (Table 1). These fragments were submitted to BLAST and used for the design of shorter fragments for the multiplex PCR. Each short fragment was selected according to its specificity, fidelity, and predicted melting temperature. The short fragment primers were designed using the online Primer 3 software (http://bioinfo.ut.ee/primer3-0.4.0/) [8]. Table 1 is presenting details of all primers from the long and the short fragments from the respective target gene for each parasite species.

**Table 1:** List of primers for amplification of the long original and the short new fragments, with fragment sizes and target genes for each parasite species

Species & Gene	Primers	PCR product	Reference
*Dientamoeba fragilis*	Long fragment	886 bp	[9]
	Dienta F: 5′-TAT CGG AGG TGG TAA TGA CC-3′		
	Dienta R: 5′-CAT CTT CCT CCT GCT TAG ACG-3′		
SSU rRNA	Short fragment	114 bp	Our study
	Dienta F2: 5′-CAA ATC AGA ACG CTT AAA GTA ATT TTC-3′		
	Dienta R2: 5′-CCC CGA TTA TTC TCT TTG ATA TT-3′		
*Entamoeba histolytica*	Long fragment	167 bp	[20]
	EntaF: 5′-ATG CAC GAG AGC GAA AGC AT-3′		
	EhR: 5′-GAT CTA GAA ACA ATG CTT CTC T-3′		
SSU rRNA	Short fragment	96 bp	Our study
	EntaF2: 5′-CGA TCA GAT ACC GTC GTA GTC C-3′		
	EhR: 5′-GAT CTA GAA ACA ATG CTT CTC T-3′		
*Cryptosporidium* spp.	Long fragment	~161 bp	[21]
	Crypto F: 5′-AGT GAC AAG AAA TAA CAA TAC AGG -3′		
	Crypto R: 5′-CCT GCT TTA AGC ACT CTA ATT TTC-3′		
SSU rRNA	Short fragment	~88 bp	Our study
	Crypto_Frag_F: 5′-GTG ACA TAT CAT TCA AGT TTC TGA CC-3′		
	Crypto_Frag_R: 5′-TAA TTC CCC GTT ACC CGT CA-3′		
	Confirmation qPCR-HRM	138 bp	[9]
	CrF 5′-CGC TTC TCT AGC CTT TCA TGA-3		
	CrR 5′-CTT CAC GTG TGT TTG CCA AT-3		
*Blastocystis* spp.	Long fragment	320-342 bp	[22]
	Bl_18rRNA F: 5′-AGT AGT CAT ACG CTC GTC TCA AA-3′		
	Bl_18rRNA R: 5′-TCT TCG TTA CCC GTT ACT GC-3′		
SSU rRNA	Short fragment	82 bp	Our study
	Bl_18rRNA_F1: 5′-GCA GTA ACG GGT AAC GAA GAA-3′		
	Bl_18rRNA_R1: 5′-TGC TGC CTT CCT TGG ATG T-3′		
*Giardia intestinalis*	Long fragment	~432 bp	[23]
	GDHiF: 5′-CAG TAC AAC TCY GCT CTC GG-3		
	GDHiR: 5′-GTT RTC CTT GCA CAT CTC C-3′		
gdh	Short fragment	~133 bp	Our study
	Giar-GDH-F3: 5′-GGC AAG AAC RTC AAG TGG-3′		
	Giar-GDH-R2: 5′-TTG TCC TTG CAC ATC TCC TC-3′		

**Fig. 1:**
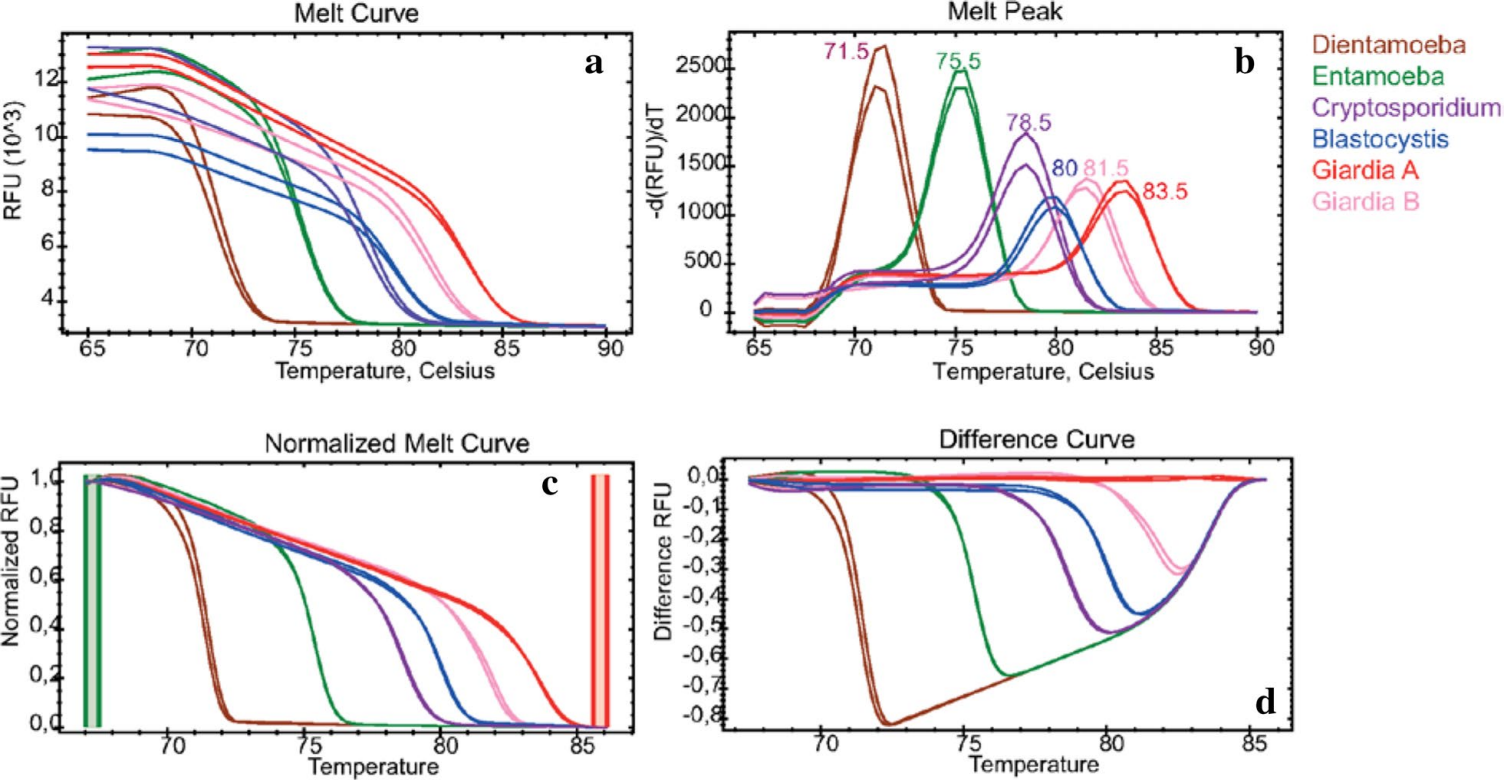
Melting curves **a**, melting peaks **b**, normalized melting peaks **c**, and differential curves **d** of reference plasmids of *Blastocystis* spp.*, Cryptosporidium* spp.*, D. fragilis, E. histolytica, G*. *intestinalis* assemblage B and *G*. *intestinalis* assemblage A. The following melting temperatures were observed: 71.5 °C for *D. fragilis* (brown), 75.5 °C for *E. histolytica* (green), 78.5 °C for *Cryptosporidium* spp. (lila), 80 °C for *Blastocystis spp.* (blue), 81.5 °C for *G. intestinalis* assemblage B (light red), and 83.5 °C for *G. intestinalis* assemblage A (red). The distance between the melting curves is well specified in the difference curve graph **d**

The 5-plex qPCR is specifically differentiating *Blastocystis* spp. (*Blastocystis*)*, Cryptosporidium* spp. (*Cryptosporidium*)*, D. fragilis* (*Dientamoeba*), *E. histolytica* (*Entamoeba*), *G. intestinalis* assemblage A (*Giardia assemblage A*)*, and G. intestinalis* assemblage B (*Giardia assemblage B*) through the melting temperature of their selected and amplified fragment (Fig. 1). We also used individual primer sets in a single-plex run for the identification of each parasite species.

**Fig. 2:**
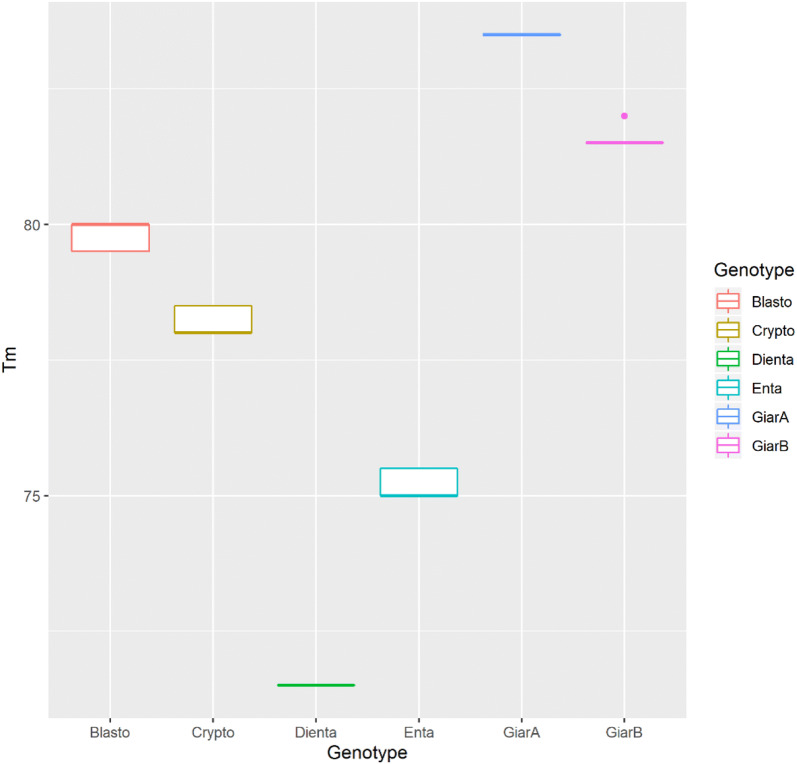
Boxplots of the melting temperatures (Tm) of *Blastocystis* spp. (red)*, Cryptosporidium* spp. (brown)*, D. fragilis* (green)*, E. histolytica* (turquoise)*, Giardia intestinalis* assemblage B (pink) and *Giardia intestinalis* assemblage A (blue). The box indicates the likely range of melting temperature variation. The melting temperature ranges were 71.5–71.5 °C for *Dientamoeba*, 75–75.5 °C for *Entamoeba*, 78–78.5 °C for *Cryptosporidium*, 79.5–80 °C for *Blastocystis*, 81.5–82 °C for *Giardia* assemblage B, and 83–83.5 °C for *Giardia* assemblage A. The melting temperature difference (∆Tm) between the closest melting curves were 3.7 °C (*Dientamoeba*/*Entamoeba*), 3.03 °C (*Entamoeba*/*Cryptosporidium*), 1.61 °C (*Cryptosporidium*/*Blastocystis*), 1.67 °C (*Blastocystis*/*Giardia* assemblage B) and 2 °C (*Giardia* assemblage B/A)

Interestingly the plasmids were presenting the same melting peak in both multiplex and single-plex runs. The melting temperature (Tm) values were 71.50 ± 0.00 °C (*Dientamoeba*), 75.20 ± 0.25 °C (*Entamoeba*), 78.23 ± 0.25 °C (*Cryptosporidium*), 79.84 ± 0.23 °C (*Blastocystis*), 81.51 ± 0.08 (*Giardia* assemblage B) and 83.50 ± 0.00 °C (*Giardia* assemblage A) (Fig. 2).

### Efficiency and limit of detection (LOD) of the assay

The sensitivity of the multiplex was tested by amplifying individually tenfold serial dilutions of plasmids containing copies (≤ 10^7^ copies) of the respective targeted gene fragment each. The multiplex assay detected target gene numbers as low as 100 copies/µl (for *Giardia* assemblage B) or even ten copies/µl (for *Blastocystis*, *Cryptosporidium*, *Dientamoeba*, *Entamoeba*, and *Giardia assemblage A*). The ranges of efficiency, slope, and R^2^ were 95.77 to 103.11%, − 3.2497 to − 3.4277 and 0.9942 to 0.9998 respectively (Table 2).

**Table 2:** Multiplex melting temperatures (Tm), specificities and limits of detection (LOD)

Species	Tm (Tm range) (°C)	Efficiency (%)	Slope	R^2^	LOD (Min–Max) copy/µl
*Dientamoeba fragilis*	71.50 ± 0.00 (71.5–7 1.5)	98.97	− 3.3468	0.9975	11.26 (7.03–25.15)
*Entamoeba histolytica*	75.20 ± 0.25 (75.0–75.5)	97.05	− 3.3948	0.9998	30.08 (18.02–69.13)
*Cryoptosporidium* spp.	78.23 ± 0.25 (78.0–78.5)	103.11	− 3.2497	0.9988	8.78 (6.06–15.99)
*Blastocystis* spp.	79.84 ± 0.23 (79.5–80.0)	97.60	− 3.3808	0.9974	10.95 (7.08–22.66)
*Giardia* intestinalis assemblage B	81.51 ± 0.08 (81.5–82.0)	95.77	− 3.4277	0.9969	20.92 (15.60–37.15)
*Giardia intestinalis* assemblage A	83.50 ± 0.00 (83.5–83.5)	96.76	− 3.4022	0.9942	20.00 (15.24–33.62)

Further dilutions were prepared (between 200 and 1 copy/µl) to determine the LOD for each parasite. The calculated LODs at 95% confidence for the multiplex method using probit analysis varied from 8.78 (6.06–15.99) copies/µl for *Cryptosporidium* to 30.08 (18.02–69.13) copies/µl for *Entamoeba* (Table 2).

The Welch’s unequal variances *t* test showed that all Tm’s were significantly different from each other as in the predicted melting, with ΔTm (between the closest Tms) value of 3.7 °C (*Dientamoeba*/*Entamoeba*), 3.03 °C (*Entamoeba*/*Cryptosporidium*), 1.61 °C (*Cryptosporidium*/*Blastocystis*), 2.67 °C between (*Blastocystis*/*Giardia* assemblage B), and 1.99 °C (*Giardia* assemblage B/*Giardia* assemblage A) (Table 2).

### Sample genotyping and multiplex specificity

**Fig. 3:**
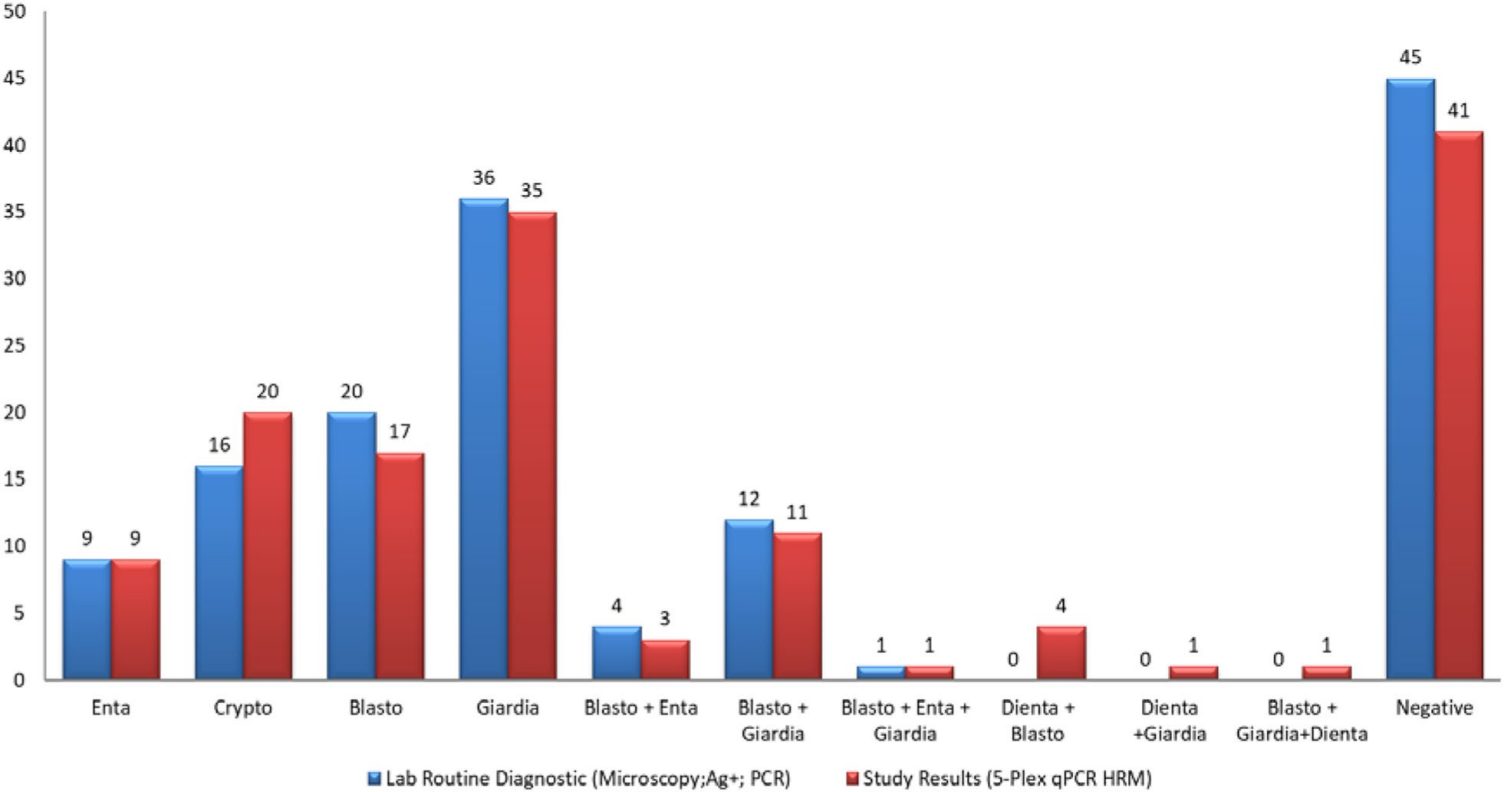
Multiplex (red) results of 143 samples as compared to routine diagnostics (blue) (microscopy (all parasites), immunoassay (*Giardia* and *Entamoeba*), FRET-qPCR analysis (*Blastocystis, Cryptosporidium, E. histolytica,* and *G*. *intestinalis*). The multiplex results of 137 samples (95.8%) matched with the routine diagnostics. The multiplex method detected four (2.8%) more *Cryptosporidium* samples and six (4.2%) samples were positive for *D*. *fragilis*

The discriminatory power of the multiplex PCR was tested using 143 DNA isolates from our routine diagnostic laboratory archive. We tested blinded samples using the multiplex method, and the results were compared to previous diagnostic results obtained through microscopy, immunoassays, and/or FRET-based qPCR. The DNA of *Entamoeba coli, Entamoeba hartmanni, Iodamoeba buetschlii* and 13 other organisms listed in the methods section was included in the tests to confirm the specificity of the method. The negative samples included parasite-free human stool samples and *Entamoeba dispar* samples. Figure 3 juxtaposes the multiplex PCR results with the original results from the routine diagnostic unit. Up to 95.8% (137) of our results were matching with the routine diagnostics. The 5-Plex method was able to detect four (2.8%) more *Cryptosporidium* infections, which were confirmed by DNA sequencing and also with Verweij et al. primers (Table 1) [9], to be *C. parvum*, and six (4.2%) samples were found to be positive for *D. fragilis*. Details on non-concordant samples with (8.4%) and without (4.2%) *Dientamoeba* data are presented in Table 3.

**Table 3:** List of non-concordant from the 143 samples with their results in both routine diagnostics and the developed 5-Plex-PCR

Samples	Lab routine diagnostic^a^	5-Plex-PCR	Percentage (%)
20	*Blastocystis*	*Blastocystis *+ *Dientamoeba*	4.19% due to *Dientamoeba*
30	*Blastocystis + Giardia*	*Blastocystis*+ *Giardia*+ *Dientamoeba*	
33	*Blastocystis*	*Blastocystis + Dientamoeba*	
61	*Blastocystis*	*Blastocystis + Dientamoeba*	
72	*Blastocystis*	*Blastocystis + Dientamoeba*	
120	*Giardia*	*Giardia + Dientamoeba*	
35	*Blastocystis + Entamoeba*	*Blastocystis*	4.19% non-concordant
6	Negative	*Cryptosporidium*	
48	Negative	*Cryptosporidium*	
49	Negative	*Cryptosporidium*	
80	Negative	*Cryptosporidium*	
119	*Giardia*	Negative	

^a^*Dientamoeba* was not included in the laboratory routine diagnostics tests

The selected primer sets were highly specific for the target species, and each parasite species was well differentiated by the individual primer set and also by the 5-plex primers mix. The non-pathogenic *amoeba* species *E. dispar Entamoeba coli, Entamoeba hartmanni, Iodamoeba buetschlii* and the 13 organisms listed in the section Materials and methods tested negative with our designed primer sets.

**Fig. 4:**
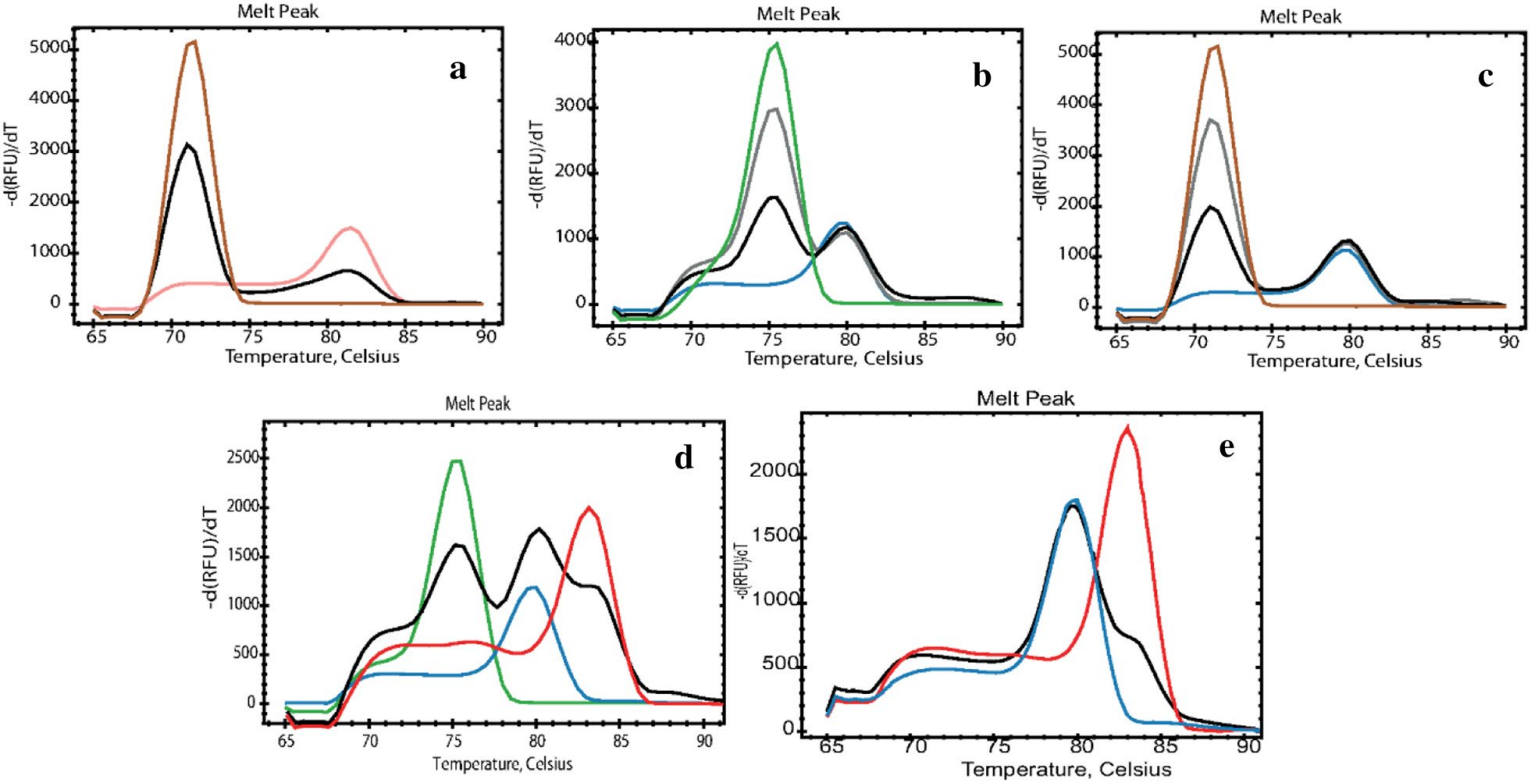
Melting peaks of mixed infection samples presenting 4 cases of double infection and one case of triple infection. All qPCR runs were done with the 5 primers mix and one color is attributed to each parasite species and the samples (black and grey): *Dientamoeba* (brown)/*Giardia B* (pink) **a**, *Entamoeba* (green)/*Blastocystis* (Blue) **b**, *Dientamoeba*/*Blastocystis***c**, *Entamoeba*/*Blastocystis*/*Giardia* assemblage A (red) **d**, and *Blastocystis/Giardia* assemblage A **e**. Singleplex runs were done with the target primers to confirm each mixedinfection

The following cases of double and triple infections were identified within the studied samples: *Dientamoeba*/*Blastocystis* (n = 5), *Dientamoeba*/*Giardia* (n = 1), *Entamoeba*/*Blastocystis* (n = 4), *Blastocystis*/*Giardia* (n = 12), and *Blastocystis*/*Entamoeba*/*Giardia* (n = 1) Fig. 4. Each mix infection was confirmed by the relative single-plex runs using the target plasmids as reference.

## Discussion

PCR and real-time PCR (qPCR) are nowadays well-established in routine diagnostics for fecal parasites. As compared to microscopy, PCR has the advantage to be fast, more sensitive, easy to perform, and not to require a long period of training, as commonly necessary for a good microscopist. PCR also has the advantage of detecting and quantifying multiple pathogens within one reaction.

Although PCR and qPCR are popular in-house testing methods for parasitological stool diagnostics, they are, in most cases, only used as a complementary method to microscopy and/or immunofluorescent analysis [9, 10].

In this study, we developed a multiplex real-time PCR (5-plex qPCR) followed by high resolution melting (HRM) curve analysis targeting specifically protists associated with diarrhea in humans. The five targeted protozoa were *Blastocystis* spp., *Cryptosporidium* spp., *Dientamoeba fragilis*, *Entamoeba histolytica*, and *Giardia intestinalis*.

To our knowledge, this method is the first non-probe-based multiplex targeting up to 5 human diarrheal parasites with well-separated melting temperatures (∆Tm > 1.5 °C). Lalonde and Gajadhar (2011) previously developed PCR melting curve analysis method targeting 8 coccidian oocysts (*Cyclosopora cayetanensis*, *C. parvum*, *Cryptosporidium muris*, *Toxoplasma gondii*, *Eimeria bovis*, *Eimeria acervulina*, *Isospora suis*, and *Sarcocystis cruzi*), with eight Tm’s ranging between 81.66 and 87.19 °C. In this case, a sample with multiple parasites would be difficult to screen using the melting peaks. Our 5-Plex, however, could be used for the screening of six different types of mixed infection samples since our melting peaks are well separated (Fig. 4). Presently, our assay is the only 5-Plex assay currently available as other multiplex tests for diarrheal parasites target either two [11], three [1, 9, 12, 13], or maximal four [14, 15] parasites.

Our novel approach harnesses well-differentiated melting temperatures (ΔTm ≥ 1.6 °C, Fig. 1), which allows the simultaneous detection and identification of all five parasites including the assemblages A and B of *Giardia*. The fact that our multiplex method is detecting more *Cryptosporidium* species (four) than established standard protocols suggests that our assay may be more sensitive as compared to the standard diagnostic qPCR method for *C. parvum*. Our method identified the same number of *Blastocystis*-positive samples as compared to established diagnostic methods used at our institute. However, four *Blastocystis samples, one mixed Blastocytis *+* Giardia sample, and one Giardia* sample were also positive for *D. fragilis*, highlighting the great advantage of our multiplex method in detecting co-infections (Table 3). The ability to simultaneously detect the five relevant parasites in a single reaction could drastically reduce the costs of parasitological stool diagnostics. Indeed, our assay is inexpensive (approximatively 6.65 USD per sample including DNA extraction) as compared to other methods (12 USD for 4 targets, Stark et al. [15]) and can be performed within 1 h 25 min. The method is also quantitative and sensitive, with *Cryptosporidium* presenting the highest sensitivity (8.78 copy/µl) and *E. histolytica* presenting the lowest sensitivity (30.08 copy/µl). We used EvaGreen which seems to be more suitable for multiplex PCR applications as compared to other dyes that are either expensive or may inhibit PCR [11, 16–18].

The limitation of our 5-plex method is the detection of mixed infections with close melting temperatures (ΔTm) and one dominant parasite infection. In such a case, only the melting peak of the major parasite would be identified.

However, this limitation is compensated by the possibility to use individual primer sets in single-plex runs to confirm and identify suspected mixed infections.

## Conclusions

We developed a fast, efficient, cost-effective and easy-to-perform 5-plex qPCR-HMR assay for simultaneous detection of *Cryptosporidium* spp., *E. histolytica,* both *G. intestinalis* assemblages (A and B), *Blastocystis* spp., and *D. fragilis* in stool samples. Moreover, the assay is suited for diagnosing mixed infections and could be expanded to widen its diagnostic potential by the inclusion of further intestinal protozoans (such as *Entamoeba coli*). It is also adaptable for epidemiological surveys and could contribute significantly to the improvement of patient management and infection control. However, the developed method still requires interlaboratory validation before its implementation in routine diagnostics.

## Methods

### Samples and DNA extraction

The method was evaluated using DNA from 143 stool samples submitted to the Institute of Specific Prophylaxis and Tropical Medicine at the Medical University of Vienna between 2011 and 2018 for diagnostic analysis. The standard diagnostic test of the study samples at our institute included microscopic analysis of stool for all intestinal parasites, immunoassay analysis (Antigens) for *G. intestinalis* and *E. histolytica,* and FRET-based qPCR (DNA) for *Blastocystis* spp., *C. parvum, C. hominis, E. histolytica, E. dispar* and *G. intestinalis.* All samples were anonymized. The Qiagen kit for stool DNA extraction (Qiagen GmbH, Vienna, Austria) was used for the samples’ DNA extraction according to manufacturer instructions.

### Target selection and primer design

A fragment of the 18S small subunit ribosomal RNA (SSU rRNA) gene (*Blastocystis* spp.*, E. histolytica, D. fragilis, Cryptosporidium* spp.) and a fragment of the NADP-dependent glutamate dehydrogenase (gdh) gene (*G. intestinalis*) were selected as targets for the assay due to their frequent use in the literature (Table 1) and were tested with one or two samples of each species (positive samples from the routine diagnostic lab) to confirm their effectiveness. The primers of *Cryptosporidium* and *Blastocystis* were selected to cover all species of these parasites as there are many pathogenic species in these genera. As *D*. *fragilis* was not included in the routine diagnostics at our institute, the long fragment of one of the positive sample (using Verweij et al. primers [9]) was sequenced by Eurofins MWG Synthesis GmbH (Ebersberg, Germany) to confirm the species before designing the short fragment primers.

New forward and/or reverse primers were designed for each parasite to target a shorter fragment with a melting temperature matching with those of the other parasites for a multiplex qPCR-HRM design (Table 1). All primers were designed using the Primer3 online tool (http://bioinfo.ut.ee/primer3-0.4.0/) and checked for specificity using basic local alignment search. We aligned the sequences with the BioEdit 7.2 Sequence Alignment Editor and produced predictive melting profiles of all fragments using the uMelt BATCH online tool (https://www.dna.utah.edu/umelt/umelt.html) before primers synthesis by Eurofins MWG Synthesis GmbH (Ebersberg, Germany).

### PCR and HRM assay

The long fragments were amplified in the same conditions with 0.5 µM primers and the following amplification program: 45 cycles of 95 °C for 15 s, 60 °C for 30 s and 68 °C for 30 s. The PCR products were resolved in 2% agarose gels and visualized by MIDORI Green Advance (Biozym Scientific GmbH, Hess Oldendorf; Germany) staining.

The multiplex PCR was performed in a volume of 20 µl containing primers, 1 × SsoFast EvaGreen Supermix (BioRad, Hercules, CA) and 4 µl DNA (≤ 20 ng/µl). Samples with DNA concentration > 20 ng/µl were diluted 10 times and retested. The optimized primers concentrations were 0.25 µM of forward and reverse primers for *D. fragilis* and *E. histolytica*, and 0.15 µM forward and reverse primers for *Cryptosporidium* spp., *Blastocystis* spp., and *G. intestinalis*.

The PCR was performed on a CFX Connect real-time PCR detection system (BioRad Laboratories, Inc., Singapore) with an initial denaturation step at 95 °C for 3 min, followed by 40 cycles of 95 °C for 5 s, 57 °C for 3 s and 68 °C for 5 s. The PCR products were subjected to the following melting program: denaturation at 95 °C for 1 min, cooling to 65 °C (held for 30 s) and continuous heating at 0.5 °C/s with fluorescence acquisition from 65 °C to 90 °C.

### HRM plasmids preparation and sequencing

The short fragments of each parasite were modified and inserted into pET17b through EcoRI/XhoI (Fermentas) restriction sites. The ligated plasmids were used to transform competent XL-1 Blue *E*. *coli* competent bacteria. Selected positive clones were cultured, the plasmids purified and sequenced by Eurofins MWG Synthesis GmbH (Ebersberg, Germany). The sequencing data were analyzed using Vector NTI.10 (Invitrogen) software, and the sequences were checked by using the Basic Local Alignment Search Tool (Nucleotide BLAST, https://blast.ncbi.nlm.nih.gov/Blast.cgi) to confirm their identity.

### Assay specificity and sensitivity

We evaluated the method specificity in situ with negative (for all tested protozoans included in this study) stool samples and mixed infections samples, and also with DNA (≤ 20 ng/µl) from the following organisms (using identical PCR conditions): *Toxoplasma gondii, Leishmania infantum, Trypanosoma brucei, Trypanosoma cruzi, Babesia divergens, Enterocytozoon bieneusi, Enzephalitozoon cuniculi, Pneumocystis jirovecii, Echinococcus granulosus, Strongyloides stercoralis, Dirofilaria repens, Toxocara canis, Entamoeba dispar, Entamoeba coli, Entamoeba hartmanni, Iodamoeba buetschlii* and *Ascaris suum*. The assay was performed in duplicate with each DNA sample.

The limit of detection (LOD) for each parasite was determined individually under the conditions of the multiplex PCR. The LOD was defined as the measured concentration producing at least 95% positive replicates [19].

The LOD was assessed by amplifying seven different concentrations (120, 80, 40, 20, 10, 5 and 1 copies/µl for *Blastocystis* spp., *Cryptosporidium* spp. and *D. fragilis*; 160, 120, 80, 40, 20, 10 and 5 copies/µl for *E. histolytica* and *G. intestinalis*) of each plasmid, in twenty (20) replicates on two separate occasions.

The total proportion of positive tests were recorded and subjected to probit regression analysis using R version 3.4.2 (2017-09-28) via RStudio version Version 1.1.383. Similarly, the boxplots of the melting temperatures (Tm) were also produced using R via the RStudio version. The Welch’s unequal variances t-test was used to compare the difference between arithmetic means of the respective Tm of the amplicons of all five parasite species using R.

## Data Availability

The datasets used and/or analyzed during the current study are available from the corresponding author on reasonable request.

## Abbreviations

BLAST: Basic local alignment search tool
DNA: Deoxyribonucleic acid
FRET: Fluorescence resonance energy transfer
HRM: High resolution melting
LOD: Limit of detection
PCR: Polymerase chain reaction
Tm: Melting temperature

## References

[1] Laude A, Valot S, Desoubeaux G, Argy N, Nourrisson C, Pomares C (2016). Is real-time PCR-based diagnosis similar in performance to routine parasitological examination for the identification of Giardia intestinalis, *Cryptosporidium parvum*/*Cryptosporidium hominis* and *Entamoeba histolytica* from stool samples? Evaluation of a new commercial multiplex PCR assay and literature review. Clin Microbiol Infect..

[2] Norman FF, Comeche B, Chamorro S, Pérez-Molina JA, López-Vélez R (2020). Update on the major imported protozoan infections in travelers and migrants. Future Microbiol..

[3] Innes EA, Chalmers RM, Wells B, Pawlowic MC (2020). A one health approach to tackle cryptosporidiosis. Trends Parasitol..

[4] Haque R (2007). Human intestinal parasites. J. Heal Popul Nutr..

[5] Sánchez A, Munoz M, Gómez N, Tabares J, Segura L, Salazar Á (2017). Molecular epidemiology of giardia, *Blastocystis* and *Cryptosporidium* among indigenous children from the colombian Amazon basin. Front Microbiol..

[6] Stensvold CR, van der Giezen M (2018). Associations between gut microbiota and common luminal intestinal parasites. Trends Parasitol..

[7] Garcia LS (2016). Dientamoeba fragilis, one of the neglected intestinal protozoa. J Clin Microbiol..

[8] Untergasser A, Cutcutache I, Koressaar T, Ye J, Faircloth BC, Remm M, et al. Primer3 Input. Whitehead Inst. Biomed. Res. 2017. http://primer3.ut.ee/.

[9] Verweij JJ, Blangé RA, Templeton K, Schinkel J, Brienen EAT, Van Rooyen MAA (2004). Simultaneous detection of *Entamoeba histolytica*, *Giardia lamblia*, and *Cryptosporidium parvum* in fecal samples by using multiplex real-time PCR. J Clin Microbiol.

[10] Verweij JJ, Rune Stensvold C (2014). Molecular testing for clinical diagnosis and epidemiological investigations of intestinal parasitic infections. Clin Microbiol Rev Am..

[11] Jothikumar N, Da Silva AJ, Moura I, Qvarnstrom Y, Hill VR (2008). Detection and differentiation of *Cryptosporidium hominis* and *Cryptosporidium parvum* by dual TaqMan assays. J Med Microbiol.

[12] Haque R, Roy S, Siddique A, Mondal U, Rahman SMM, Mondal D (2007). Multiplex real-time PCR assay for detection of *Entamoeba histolytica*, *Giardia intestinalis*, and *Cryptosporidium* spp.. Am J Trop Med Hyg..

[13] Parčina M, Reiter-Owona I, Mockenhaupt FP, Vojvoda V, Gahutu JB, Hoerauf A (2018). Highly sensitive and specific detection of *Giardia duodenalis*, *Entamoeba histolytica*, and *Cryptosporidium* spp. in human stool samples by the BD MAX™ Enteric Parasite Panel. Parasitol Res.

[14] Van Bruijnesteijn Coppenraet LES, Wallinga JA, Ruijs GJHM, Bruins MJ, Verweij JJ (2009). Parasitological diagnosis combining an internally controlled real-time PCR assay for the detection of four protozoa in stool samples with a testing algorithm for microscopy. Clin Microbiol Infect..

[15] Stark D, Al-Qassab SE, Barratt JLN, Stanley K, Roberts T, Marriott D (2011). Evaluation of multiplex tandem real-time PCR for Detection of *Cryptosporidium* spp, *Dientamoeba fragilis*, *Entamoeba histolytica*, and *Giardia intestinalis* in clinical stool samples. J Clin Microbiol..

[16] Eischeid AC (2011). SYTO dyes and EvaGreen outperform SYBR Green in real-time PCR. BMC Res Notes..

[17] Bustin SA, Benes V, Garson JA, Hellemans J, Huggett J, Kubista M (2009). The MIQE guidelines: minimum information for publication of quantitative real-time PCR experiments. Clin Chem.

[18] Marangi M, Giangaspero A, Lacasella V, Lonigro A, Gasser RB (2015). Multiplex PCR for the detection and quantification of zoonotic taxa of Giardia, *Cryptosporidium* and Toxoplasma in wastewater and mussels. Mol Cell Probes.

[19] Forootan A, Sjöback R, Björkman J, Sjögreen B, Linz L, Kubista M (2017). Methods to determine limit of detection and limit of quantification in quantitative real-time PCR (qPCR). Biomol Detect Quantif. Elsevier GmbH.

[20] Hamzah Z, Petmitr S, Mungthin M, Leelayoova S, Chavalitshewinkoon-Petmitr P (2006). Differential detection of *Entamoeba histolytica*, *Entamoeba dispar*, and *Entamoeba moshkovskii* by a single-round PCR assay. J Clin Microbiol.

[21] Lalonde LF, Gajadhar AA (2011). Detection and differentiation of coccidian oocysts by real-time PCR and melting curve analysis. J Parasitol.

[22] Poirier P, Wawrzyniak I, Albert A, El Alaoui H, Delbac F, Livrelli V (2011). Development and evaluation of a real-time PCR assay for detection and quantification of *Blastocystis parasites* in human stool samples: prospective study of patients with hematological malignancies. J Clin Microbiol.

[23] Read CM, Monis PT, Thompson RCA (2004). Discrimination of all genotypes of Giardia duodenalis at the glutamate dehydrogenase locus using PCR-RFLP. Infect Genet Evol..

